# Silver nanoparticles (AgNPs) based selective colorimetric sensor for the detection of D-arabinitol in a urine sample

**DOI:** 10.55730/1300-0527.3482

**Published:** 2022-06-01

**Authors:** Yuni RETNANINGTYAS, Siswandono SISWODIHARDJO, Ari SATYA NUGRAHA

**Affiliations:** 1 Faculty of Pharmacy University of Jember, Jember, Indonesia; 2 Department of Pharmaceutical Chemistry, Faculty of Pharmacy University of Airlangga, Surabaya, Indonesia

**Keywords:** D-arabinitol, colorimetry, silver nanoparticles, candidiasis, reduction

## Abstract

D-arabinitol is a sugar alcohol that is a typical metabolite of Candida species. The hydroxy group owned by D-arabinitol can function as a reducing agent that can reduce Ag^+^ to AgNPs. The resulting colloid is further stabilized by the addition of a capping agent. The capping agent used in this study was a combination of chitosan 1%(w/w) and PEG 6000 1% (w/w) (2:1) v/v. The formation of AgNPs causes an increase in the surface plasmon resonance spectra of the colloid solution at a wavelength of 430 nm with a slight blue shift. At the same time, the color of the solution changes from colorless to yellow colloid so that the absorbance can be observed using a spectrophotometer. The change in the absorbance value of the colloid produced is proportional to the concentration of D-arabinitol added, so that the amount of D-arabinitol in the sample can be quantified. Under optimum conditions, the resulting method shows good linearity with an R^2^-value of 0.9979 in the concentration range of D-arabinitol 100–2000 μM and Vxo value is 4.30%. The detection limit is 115.11 μM and the quantification limit is 383.70 μM. The repeatability (%RSD) is 0.78–1.94 and the % recovery in addition to urine samples is 93.27–103.70. This method is quite selective for other components present in urine. The analysis time is short, the number of samples required is only small, and does not require an organic solvent, so this method has the potential to be used in determining the levels of D-arabinitol in urine samples.

## 1. Introduction

Invasive candidiasis is a serious health concern, it is a fungal infection caused by several Candida species, the most common of which is Candida albicans. According to the clinical data, invasive fungal infections have been swiftly increasing lately. Despite the development of novel antifungal therapeutic strategies, the high mortality due to fungal infections is still considered a critical matter. Rapid and unambiguous diagnosis of these infections at their earliest stages is critical for successful treatment [1]. The determination of D-arabinitol concentration is one of the most advantageous and convenient methods of diagnosing Candida sp infections. Without a doubt, the most significant advantage of this concentration determination is the ability to make an early diagnosis, allowing for immediate treatment initiation and patient protection from unnecessary complications. [2]

D-arabinitol is a sugar alcohol with 5 hydroxy groups which is a typical and pathogenic metabolite of several Candida species such as *C. albicans, C. parapsilosis*, and *C. tropicalis*. D-arabinitol is produced from carbohydrate intake when yeast grows rapidly under anaerobic conditions in the small intestine. D-arabinitol is a natural metabolite produced by human cells in almost equal amounts. The normal level of D-arabinitol in serum is about 0–5 μM [3] while that in urine is about 55.0–950.0 μM [4]. D-arabinitol serum and urine levels will increase if Candida fungi multiply in the organism [5]. Human cells, naturally produce not only D-arabinitol but also L-arabinitol in almost the same amount. While fungi of the Candida family only produce D-arabinitol, therefore, an increase in the concentration of D-arabinitol in body fluids is a diagnostic indication of invasive candidiasis. To make D-arabinitol a candidiasis diagnostic, an analytical method is needed that can detect D-arabinitol accurately, selectively, easily, and quickly.

The determination of the ratio of D and L-arabinitol can be done by several analytical methods, including gas chromatography [5–9]. However, the detection of D-arabinitol with this method has a weakness because D-arabinitol belongs to the xylitol group so the peaks marked as D-arabinitol can come from adonitol, D-arabinitol, and L-arabinitol, so to separate these compounds requires a column containing-cyclodextrin [6]. In addition, D-arabinitol has inactive electrochemical characteristics and weak UV-Visible absorption. Therefore, before the analysis, it is necessary to do derivatization first [8], [1]. In addition to using the chromatographic method, the determination of D-arabinitol levels can also be carried out using the enzymatic method [10], the fluorometric enzymatic method for determining arabinitol in human serum [11], cyclic voltammetry based on the electrochemical oxidation of D-arabinitol [12], The pezomicrogravimetric chemosensor using molecularly imprinted polymer (MIP) and field-effect transistor chemosensor with extended gate using molecularly imprinted polymer (MIP) [2]. These techniques necessitate the use of relatively expensive laboratory instrumentation and trained personnel even though it is accurate. As a result, many hospitals, physicians, and most importantly, patients at home are unable to use them for diagnostic purposes. Consequently, there is a clear need to develop a low-cost, simple method for determining D-arabinitol enantioselectivity.

D-arabinitol and L-arabinitol are isomers that both have the same molecular formula and molecular weight but have different structural formulas and physical properties. D-arabinitol and L-arabinitol are both present in the urine in the form of enantiomers so their separation requires a specific separation process. Selective separation of these two compounds can be carried out using molecularly imprinted polymer (MIP) [13]. The synthesis of MIP to separate D and L-arabinitol in urine samples has previously been carried out, where the MIP produced has been able to specifically separate D and L-arabinitol [14]. The results of this separation allow the determination of D-arabinitol in urine separately.

Nanoparticles are very fine particles that have the size of nanometers. At this size, the physical, chemical, and biological properties of the nanoparticles differ from those of a single atom/molecule. Nanoparticle materials are currently widely used in the field of analysis, including in the field of colorimetric bioanalysis. Colorimetry is one of the analytical methods that can detect the presence of analytes with the naked eye. The most important thing in using nanoparticles in colorimetry is the selection of organic components as ligands to modify nanoparticles. When the organic molecule gives a specific response to the analyte, it will cause a change in color and UV-Vis spectrum due to the aggregation of nanoparticles or the formation of nanoparticles.

Recently, sugars and polyols analysis have been reported in AgNPs-based food samples [15], [16]. The development of this method is based on the ability of sugars and polyols commonly found in food to mediate the formation of AgNPs at room temperature. The formation of AgNPs depends on the structure and concentration of the analyte which is characterized by the formation of a yellow-colored suspension (AgNPs) where this suspension will give a colorimetric response at a fixed wavelength (AgNPs: 430 nm) [15]. In this study, D-arabinitol as an analyte is a sugar alcohol that has 5 hydroxy groups which is an isomer of Xylitol that has the potential to mediate the formation of AgNPs at room temperature. From the description stated previously, this research aims to develop a new method to detect D-arabinitol in urine more accurately, quickly, easily, and cheaply. The new method developed in this study is a sensor based on the formation of AgNPs for the detection of D-arabinitol in urine samples that have previously been separated by MIP.

## 2. Materials and methods

### 2.1. Materials

D-arabinitol (C_5_H_12_O_5_), silver nitrate (AgNO_3_, > 99%), Polyethylene glycol 6000 (PEG 6000) were purchased from Sigma-Aldrich Co. Ltd. (St. Louis, MO, United States), chitosan (C_56_H_103_N_9_O_39_), sodium hydroxide (NaOH) were purchased from Merck (Kenilworth, NJ, USA) and deionized water (DI water) was used for all experiments purchased from Brataco ( Surabaya, Indonesia).

### 2.2. Equipment

The Branson 2510 ultrasonic cleaner from Marshall Scientific (USA) was used to disperse the mixtures. Shimadzu UV-2600 UV-Vis spectrophotometer from Shimadzu (Japan) was used for absorbance measurement. EBA20-Hettich was used to centrifuge and separate the urine samples from Merck (Germany). Zetasizer Nano ZS (Malvern Instruments Ltd., Grovewood, Worcestershire, UK) with DTS nano version 7.01 software (Malvern Instruments Ltd.) was used to measure the particle size distribution of AgNPs. Shimadzu LC-MS 2020 from Shimadzu (Japan) for reversed-phase high-performance liquid chromatography (LC-MS) on a SunFire C18 with a 5 m particle size, 4.6 mm internal diameter and 250 mm length (Waters, Milford, USA) column was used as a comparison method.

### 2.3. Urine sample

The urine samples referred to in this study were from healthy children. The healthy children aged 2 to 12 years male gender who did not receive antibiotics or antifungals were acquired from volunteers in Jember, Indonesia.

### 2.4. Synthesis of MIP D-arabinitol

The following procedure was used to complete the synthesis of MIPs D-arabinitol: In 150 mL Erlenmeyer, 1 mmol of a template (D-arabinitol), 4 mmol of monomers (acrylamide), 20 mmol of cross-linker (EGDMA), and 50 mL of porogen (DMSO) were added and stirred until a homogeneous solution was formed. The initiator, 1% b/v benzoyl peroxide in chloroform, was then added and thoroughly mixed. For 15 min, this solution was purged with nitrogen gas to remove air bubbles and allow the dissolution process to run smoothly. The Erlenmeyer was then sealed and immersed in water. Polymer synthesis was carried out for 12 h at 60 °C. The polymers were then filtered out using a vacuum pump and dried in a 40 °C oven before being crushed to a particle diameter of 50 mm or less [14]. To obtain MIPs, D-arabinitol molecules were extracted from MIPs by mixing MIPs with methanol: acetic acid (4:1, v/v) solution for 60 min and repeating the process five times until no D-arabinitol was detected in the washing solution. MIP washing was then resumed with the addition of methanol. The D-arabinitol MIP extracted was then utilized to extract D-arabinitol from urine samples

### 2.5. Preparation of D-arabinitol standard solution

The stock solution of D-arabinitol 2000 M was prepared by dissolving 30.43 mg of D-arabinitol in DI water up to 10 mL. A working standard was then prepared by pipetting a certain volume of stock solution and adding DI water to 10 mL to obtain a D-arabinitol solution with a concentration of 100–2000 μM.

### 2.6. AgNPs-based assay

It is inevitable to maximize the analysis conditions to come up with the optimal analysis results. The conditions optimized in this study included the selection and composition of the capping agent. The composition of the capping agent used in the optimization was 1% Chitosan and 1% PEG 6000 with a ratio (1:1); (1:2); (2:1)% v/v. The next optimization is the comparison between the AgNO_3_ (5.17 × 10^−3^ mol L^−1^) and the capping agent. The ratio of the amount of AgNO_3_ and the capping agent used in the optimization is (1:1), (2:1), (1:2) v/v. The volumes of NaOH (5 mol L^−1^) which are used in optimation is 5, 10, 20, 30, 40, and 50 μL. The incubation temperatures used in optimation are 25, 35, 45, and 55 °C. The incubation times used in optimation are 10, 20, 30, 40, and 50 minutes, and the wavelength of measurement is 300–800 nm. The results of the optimization were then used in the synthesis of AgNPs: 250 μL of AgNO_3_ (5.17 × 10^−3^ mol L^−1^) and capping agent (1:2 v/v), and 200 μL of samples were added to 510 μL DI water, then 40 μL NaOH (5 mol L^−1^) was added to trigger the reaction. At 45 °C, the reaction was started and stirred for 30 minutes. The reaction was stopped by putting it on ice for 10 minutes and then observing it with UV-Vis spectrophotometry at 430 nm.

### 2.7. AgNPs and analytical characterizations of the sensor

The endpoint reactivity was determined by plotting the absorbance at 430 nm (A_430_) of the AgNPs plasmon band against the concentration of D-arabinitol. UV-Vis Spectra and Particle Size Analysis (PSA) were utilized to characterize AgNPs formed by D-arabinitol action. The UV-Vis spectra were recorded against a blank in the range of 300–800 nm. A dynamic light scattering (DLS) technique with the Zetasizer Nano ZS was utilized to measure the particle size in the present study carried through the PSA characterization. Linearity, LOD and LOQ, precision, and accuracy were the validation parameters to test the performance of the sensor to detect D-arabinitol at optimum conditions.

### 2.8. Urine sample analysis

The samples of the urine that has been added 500.0 μM of D-arabinitol standard approximately 200 μL that had been extracted with MIPs D-arabinitol was added with 250.0 μL of a mixture of AgNO_3_ and the capping agent (1:2) v/v then added 40 μL of NaOH (5 mol L^−1^) added DI water to 1.00 mL in a vial shake for 30 min at 45 °C. The reaction was stopped by placing the sample on an ice bath for 10 min and observed at a wavelength of 430 nm. The results of this determination are then compared with the LC-MS method. The LC-MS was conducted by using the C18 column (150 × 4.6 mm, 5 m) with the mobile phase consisting of acetonitrile and DI water in the ratio of 80:20 v/v, respectively. The flow rate was set at 0.5 mL/minutes with m/z detection at 152 and injection volume was set at 20.0 μL [14].

## 3. Results and discussion

### 3.1. Optimization condition of synthesis of AgNPs

**Figure 1:**
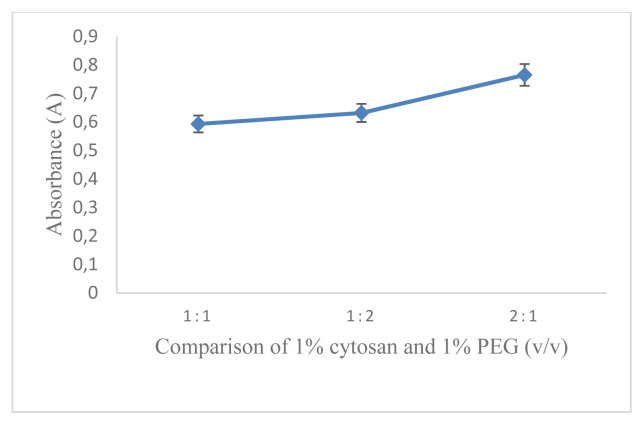
Graph correlation composition of capping agent vs. absorbance.

**Figure 2:**
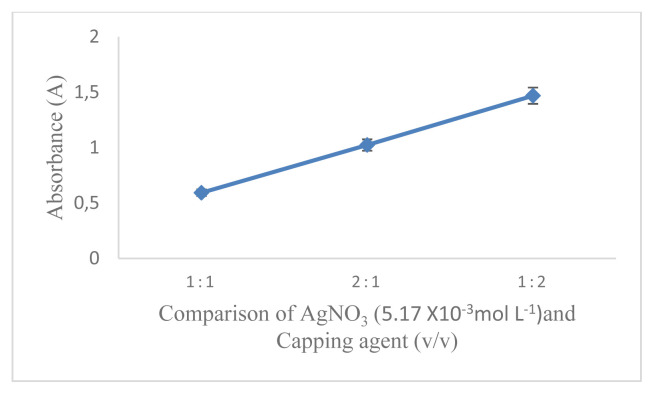
Graph correlation composition of AgNO_3_ and capping agent vs. absorbance.

**Figure 3:**
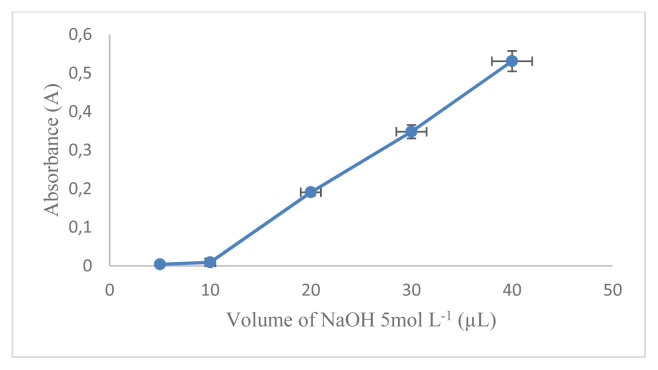
Graph correlation Volume of NaOH 5mol L^−1^ (μL) vs. absorbance.

**Figure 4:**
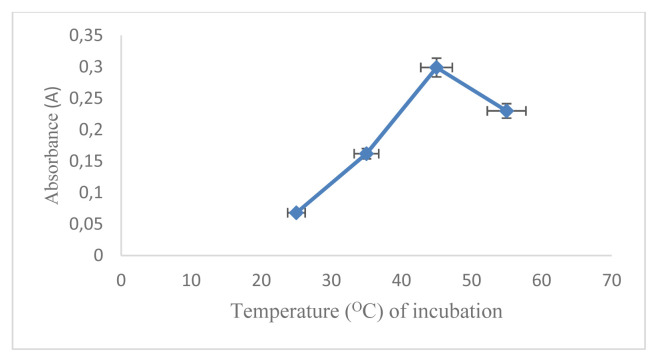
Graph correlation temperature (°C) of incubation vs. absorbance.

**Figure 5:**
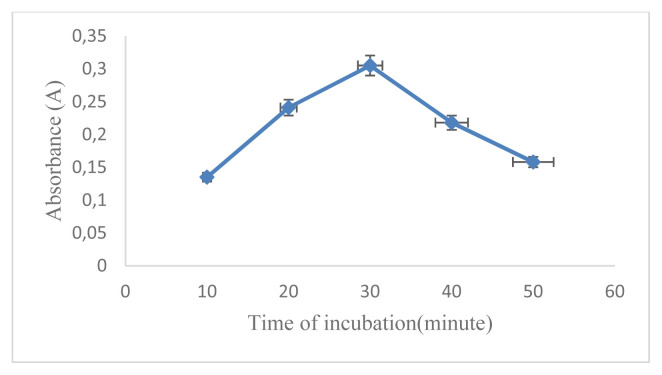
Graph correlation time of incubation (min) vs. absorbance.

The aim of the work was the development of a D-arabinitol-mediated single-pot optical assay based on AgNPs as signal transducers. AgNPs have their unique SPR band, which strongly depends on size, shape, interparticle distance, and the surrounding medium. The formation of colloidal MNPs suspensions results from the concomitant reduction of the metal source and the stabilization of the MNPs produced, at the same time their aggregation/collapse, and irreversible MNPs conformational changes with LSPR extinction band variation should be avoided [16]. Therefore, it is necessary to optimize the reaction conditions that can affect the factors mentioned above. In this study, the concentration of AgNO_3_ used was 5.17 × 10^−3^mol L^−1^ referring to previous studies [17]. The first optimized reaction condition was that the capping formula used is very influential on the formation of AgNPs and will also affect the changes in SPR. In this research, optimization has been carried out using several kinds of capping agents including citric acid, EDTA, cysteamine, cysteine, and a combination of chitosan and PEG. The optimization results show that the capping agent that can produce AgNPs with D-arabinitol as a reducing agent is a combination of 1% chitosan and 1% PEG. After determining the selected capping agent, the comparison between PEG and chitosan was optimized. The most optimal capping agent formula for the formation of AgNPs with the composition of chitosan (1%): PEG (1%) is 2:1 (v/v) as shown in (Figure 1). It is characterized by the formation of stable silver nanoparticles and has the largest absorbance value. The comparison between the amount of AgNO_3_ and the capping agent also greatly affects the AgNPs produced and the SPR value. The most optimal comparison of the AgNO_3_ and capping agent with the largest A value is 1: 2 (v/v) as shown in (Figure 2). NaOH in this reaction serves to initiate the reaction for the formation of silver nanoparticles. The amount of NaOH added to this reaction greatly affects the resulting nanoparticles. The most optimum amount of NaOH in initiating the reduction of AgNO_3_ by D-arabinitol is 40 μL (5 mol L^−1^) as shown in (Figure 3). Other factors that influence the reduction of AgNO_3_ by D-arabinitol are temperature and incubation time. The temperature and reaction time of the proposed method was determined by monitoring the decrease in the SPR absorbance as a function of temperature and time, after the colloidal solution of AgNPs was formed. The highest absorbance value was achieved after the incubation temperature reached 45 °C as shown in (Figure 4). The results of optimization of incubation time show that the increase in incubation time also causes an increase in the absorbance value. Then, the absorbance gradually decreases after incubation for more than 30 min. Based on these results, the optimal incubation time is 30 min as shown in (Figure 5).

### 3.2. AgNPs characterization

**Figure 6:**
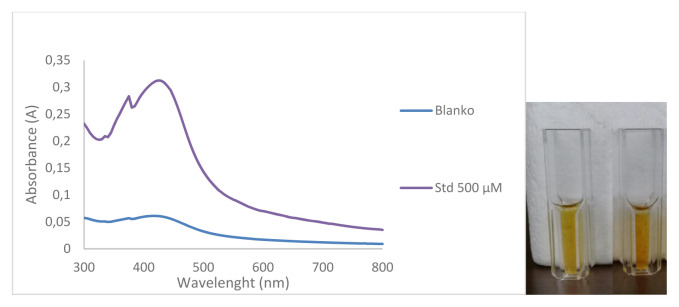
UV-Vis photometric spectra of the sample (500 μM D-arabinitol) and blank.

The characterization of the synthesized AgNPs was carried out using DLS, and UV-Vis Spectrophotometry [18]. UV-Vis spectra produced by AgNPs will give an absorbance value of 400–500 nm depending on the particle size produced, the larger the particle size of the resulting nanoparticles, the redshift (the longer the wavelength) will be [19]. The results of the analysis of the spectra of the blank solution and the sample solution using UV-Vis Spectrophotometry are shown in (Figure 6). The results of the UV-Vis spectrophotometer measurement in (Figure 6) show the formation of silver nanoparticles which is characterized by the presence of a typical absorption peak at max 400–500 nm, with a peak wavelength of 430 nm. This is in line with the research previously where the absorption spectra of silver nanoparticles formed at max 415–430 nm [20]. Figure 6 also shows the difference in the ability of the blank solution and the sample solution to reduce AgNO_3_ to form AgNPs which are characterized by different absorbance values between the blank solution and the sample solution, where at the same wavelength the absorbance value of the sample is 0.311 while the absorbance value of the blank is 0.06. This means that D-arabinitol can reduce AgNO_3_ to form AgNPs.

**Figure 7:**
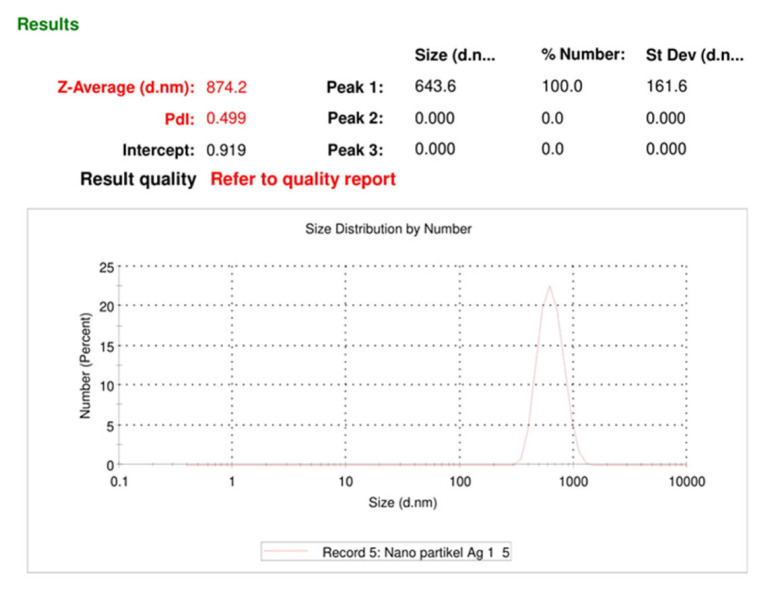
Size measurement results of AgNPs synthesized with PSA.

The results of the measurement of the particle size distribution of the sample using the DLS technique are shown in (Figure 7). Based on the results shown in (Figure 7), the value of the hydrodynamic diameter (Z-average) of the reduced Ag particles with D-arabinitol is 874.2 and the Polydispersity index (PDI) value is 0.499. The resulting AgNPs have a size that is to the theory, where a particle can be categorized as a nanoparticle if it has a size with a diameter between 1–1000 nm [21]. The PDI value of 0.499 for AgNPs indicates that these AgNPs are in moderate polydispersity conditions because PDI values between 0.08–0.7 are included in the medium polydispersity category [22]. These results indicate that D-arabinitol can reduce Ag^+^ to form AgNPs, where the AgNPs formed have been composited in the [Chitosan/PEG] composite so that the mobility of silver particles measured in PSA is analyzed as a composite [Ag/Chitosan/PEG].

### 3.3. Principle of D-arabinitol sensing

**Figure 8:**
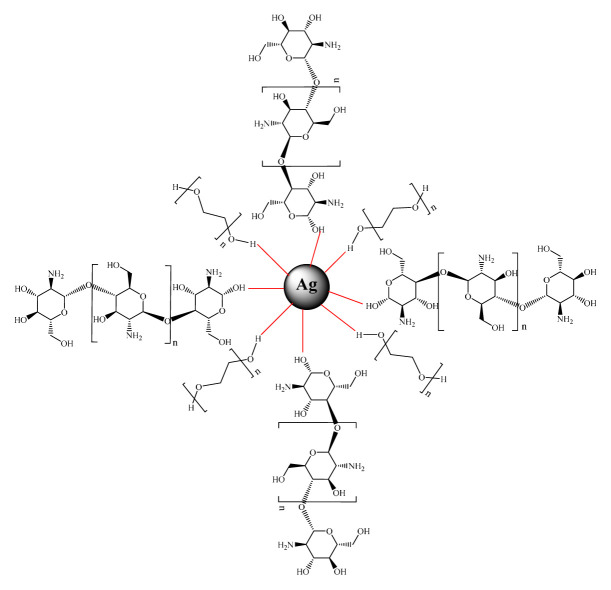
Distribution and interaction of Ag+ ions on capping agents and stabilizers.

This research aims to develop an analytical method for determining the levels of D-arabinitol mediated single-pot optical assay based on AgNPs as signal transduce which is simple, fast, sensitive, and effective. AgNPs as indeed, under the influence of electromagnetic radiation in the visible range, the electrons of surface atoms can easily move through vacant orbitals generating absorption at a particular wavelength. This phenomenon is called localized surface plasmon resonance (LSPR). The principle of determining D-arabinitol in this study is based on the formation of silver nanoparticles by chemical reduction using D-arabinitol as a reducing agent. In general, there are three important components in the chemical reduction of silver nanoparticles, namely metal precursors, reducing agents, capping agents, and stabilizers. In this study, silver metal precursors in the form of AgNO_3_ salts were used, while chitosan and PEG-6000 were used as capping agents and stabilizers of silver nanoparticles. Chitosan is known to have the ability as a good complexing compound wherein the chain there is an amine group (-NH_2_) which can interact specifically with a metal. In addition, chitosan is also a hydrophilic compound that has a hydroxyl group (-OH) wherein the presence of -OH and -NH_2_ chitosan groups can form secondary interactions through hydrogen bonds with other polymers [23]. PEG has been widely used as an effective passivating agent in the synthesis of Ag NPS and other metal nanoparticles. The hydroxyl group of PEG as a capping agent will envelop the silver nanoparticles, this happens because the surface of the AgNPs is positively charged. Stabilization of [Ag (PEG)] occurs due to the van der Waals forces between the oxygen contained in the PEG molecular structure. The distribution and interaction of Ag^+^ with capping agents (Ag/chitosan and PEG) are shown in (Figure 8).

### 3.4. Analytical characterizations of the sensor

**Figure 9:**
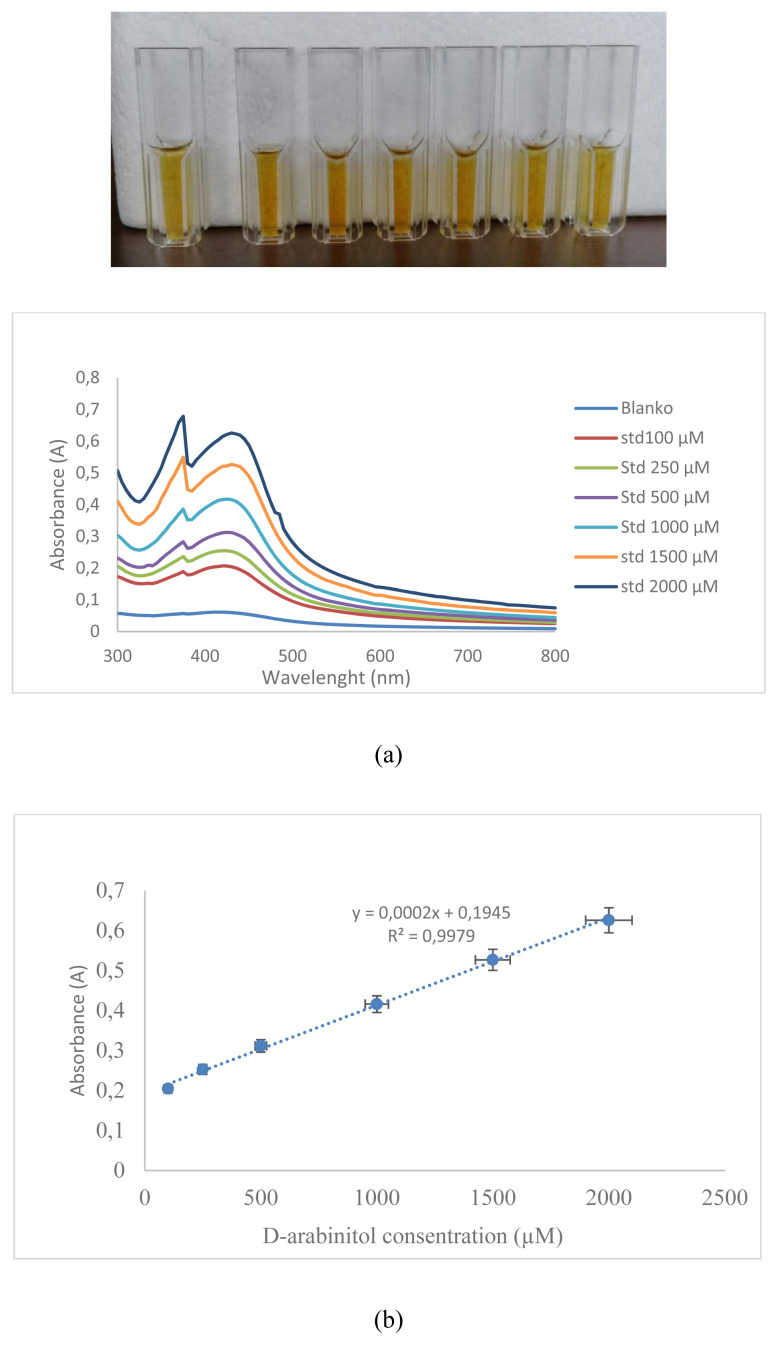
Spectra (a) and curve (b) linearity test of the method.

AgNPs were used for the determination of D-arabinitol levels under optimal analytical conditions and a calibration curve was drawn by plotting the ratio of absorbance to the concentration of D-arabinitol (Figure 9). The results of determining linearity show that the calibration curve is linear in the concentration range of 100.00 to 2000.00 μM with the regression equation y = 0.0002x + 0.1945, with R^2^ = 0.9979, and the relative process standard deviation value (Vxo) is 4.30%. This result met the requirements of the r-value > 0.99, Vxo < 5% [24]. This result confirmed the good linearity of the sensor. The resulting linearity curve gradually increases the absorbance value with increasing D-arabinitol concentration. This method’s LOD and LOQ values based on the linearity curve obtained are 115.11 and 383.70 μM, respectively. This method shows an adequate precision value with the RSD values for repeatability that is 0.78%–1.94%, the method also has been successfully developed and is also accurate with a 93.27%–103.70% recovery value. The outcome of determining the precision and accuracy of the sensor is shown in (Table 1). The result of the accuracy and precision test fulfilled the AOAC requirement [24]. The resulting calibration curve was then used to determine the level of D-arabinitol contained in the additional urine sample that had previously been separated by MIPs. These results indicate that the colorimetric sensor that has been successfully developed can detect D-arabinitol accurately and precisely. The advantage of this method compared to other previously developed methods as shown in (Table 2) is that this method can be carried out quickly, easily, and simply with accurate, precise, selective analysis results and has a wide analysis range. Another advantage of this sensor is that it is cheap because it uses very few reagents and is environmentally friendly because it does not require organic solvents.

### 3.5. Determination of D-arabinitol levels in urine samples

The method that has been developed and its performance assessed with method validation is then used to determine the levels of D-arabinitol in urine samples. The sample used here is a boy’s urine sample. The results of the next analysis were compared with the results of the analysis using the LC-MS method on the same sample. The results of determining the levels of D-arabinitol in urine samples with the developed method and the comparison method are shown in (Table 3).

Based on the results shown in Table 3 showed that the developed method could be used to determine the level of D-arabinitol in urine samples accurately and did not differ significantly when compared to the LC-MS method. The results of the statistical test with the t-test showed that the results of the measurement of the sample solution using the colorimetric method when compared with the results of the measurement using the standard method (LC-MS) had a significance value of more than 0.05 in all sample solutions (A, B, C) which are 0.792, 0.771, and 0.406, respectively. This proves that the developed method provides measurement results that were no different when compared from the results of measurements using the standard method (LC-MS) with a 95% confidence level.

## 4. Conclusion

In this study, a colorimetric sensor has been successfully developed for the determination of D-arabinitol levels in urine samples based on the formation of AgNPs. The principle of determining the level of D-arabinitol here is based on the ability of D-arabinitol to reduce AgNO_3_ to AgNPs proportional to its concentration. The AgNPs-based assay here reported results more simple, fast, cost-effective (very few reagents used), and environmentally friendly. The resulting sensor can detect D-arabinitol in the urine accurately and selectively. The results of this study currently can be an interesting alternative that can be used in clinical analysis, especially for the initial diagnosis of candidiasis.

## Figures and Tables

**Table 1:** The precision and recovery of D-arabinitol in urine addition.

Theoretical concentration (μM)*	The concentration found (μM) *	Recovery (%)	SD	RSD (%)
550	513	93.27	1.02	1.01
1620	1680	103.70	0.81	0.78
1923	1946	101.20	1.97	1.94

* 3 replication of each level

**Table 2:** Analytical methods of D-arabinitol in biological samples.

Analytical method	Advantages	Disadvantages	Analytical characterizations Reference
**GC-MS** Determination of relative amounts of D-and L-arabinitol in urine using chiral stationary phase and an electron-capture detector	Efficient online sample purification Available for routine application	Expensive Requires expensive equipment Takes a long time Requires trained personnel	Linearity range (164–1643 μM) Larsson et al. (1994)
**Colometric enzymatic assay** Determination of INT-formazan by colorimetry. INT-Formazan is formed from the reduction of p-iodonitrotetrazolium by D-arabinitol. INT-Formazan, a product of two consecutive enzymatic reactions involving (D-arabinitol dehydrogenase, and then diaphorase)	Applicable to all biological samples Available in the market in kit form (ARABINITEC-AUTO^®^)	Expensive Requires specific reagents (INT-Formazan, NADH) Less reproducible because it involves recombinant proteins	Linearity range (1–26 μM) Switchenko et al. (1994)
**Fluorometric enzymatic assay** Fluorometric determination of NADH, NADH is the product resulting from the enzymatic oxidation reaction of D-arabinitol by D-arabinitol dehydrogenase.	Applicable to all biological samples	Expensive Requires specific reagents (NADH) Less reproducible because it involves recombinant proteins	Linearity range (1.1–21.1 μM) Yeo et al. (1994)
**Cyclic voltammetry (CV)** Electrochemical oxidation of D-arabinitol	The analysis procedure is fast and simple No need for expensive laboratory equipment	Less selective Requires sample pretreatment by chromatography	Linearity range (10 μM–10 mM) LOD 1.0 μM Wang et al. (2010)
**MIP QCR sensor** Piezomicrogravimetric chemosensor using molecularly imprinted polymer (MIP)	The analysis procedure is easy and simple No need for expensive laboratory equipment High selectivity	Cannot be applied to all biological samples High detection limit	Linearity range (150–1250 μM) LOD 150.0 μM Dabrowski et al. (2016)
**MIP EG-FET sensor** Field-effect transistor chemosensor with extended gate using molecularly imprinted polymer (MIP)	The analysis procedure is easy and simple No need for expensive laboratory equipment High selectivity	Cannot be applied to all biological samples High detection limit	Linearity range (120–1000 μM) LOD 120 μM Dabrowski et al. (2016)
**MIP colorimetry sensor** Colorimetric sensor based on AgNPs formation with D-arabinitol as reducing agent	The analysis procedure is fast, easy, and simple Wide linearity range, high selectivity, accurate and precise Cost-effective (very few reagents used) Environmentally friendly (does not require an organic solvent)	Cannot be applied to all biological samples, except urine High detection limit	Linearity range (150–2000 μM) LOD 115.11 μM This work

**Table 3:** Comparison of the results of determining the levels of D-arabinitol in urine samples with the LC-MS method.

Sample	Colorimetry method (μM)	LC-MS method (μM)
A	67.55	66.81
B	100.031	98.66
C	83.05	82.10
